# Physical Health-Related Quality of Life Improves over Time in Post-COVID-19 Patients: An Exploratory Prospective Study

**DOI:** 10.3390/jcm12124077

**Published:** 2023-06-15

**Authors:** Stefan Malesevic, Noriane A. Sievi, Dörthe Schmidt, Florence Vallelian, Ilijas Jelcic, Malcolm Kohler, Christian F. Clarenbach

**Affiliations:** 1 Faculty of Medicine, University of Zurich, 8006 Zurich, Switzerland; stefan.malesevic@usz.ch; 2 Department of Pulmonology, University Hospital Zurich, 8091 Zurich, Switzerland; 3 Department of Cardiology, University Hospital Zurich, 8091 Zurich, Switzerland; 4 Department of Internal Medicine, University Hospital Zurich, 8091 Zurich, Switzerland; 5 Department of Neurology, University Hospital Zurich, 8091 Zurich, Switzerland

**Keywords:** post-COVID-19, health-related quality of life, physical health, mental health, follow-up

## Abstract

(1) Background: Ongoing symptoms after mild or moderate acute coronavirus disease 19 (COVID-19) substantially affect health-related quality of life (HRQoL). However, follow-up data on HRQoL are scarce. We characterized the change in HRQoL over time in post-COVID-19 patients who initially suffered from mild or moderate acute COVID-19 without hospitalization. (2) Methods: Outpatients who visited an interdisciplinary post-COVID-19 consultation at the University Hospital Zurich and suffered from ongoing symptoms after acute COVID-19 were included in this observational study. HRQoL was assessed using established questionnaires. Six months after baseline, the same questionnaires and a self-constructed questionnaire about the COVID-19 vaccination were distributed. (3) Results: In total, 69 patients completed the follow-up, of whom 55 (80%) were female. The mean (SD) age was 44 (12) years and the median (IQR) time from symptom onset to completing the follow-up was 326 (300, 391) days. The majority of patients significantly improved in EQ-5D-5L health dimensions of mobility, usual activities, pain and anxiety. Furthermore, according to the SF-36, patients showed clinically relevant improvements in physical health, whereas no significant change was found regarding mental health. (4) Conclusions: Physical aspects of HRQoL in post-COVID-19 patients relevantly improved over 6 months. Future studies are needed to focus on potential predictors that allow for establishing individual care and early interventions.

## 1. Introduction

Long-term health consequences after acute COVID-19 are increasingly recognized and lead to a high individual burden. Multiple organ systems may be affected and lead to variable clinical presentations, including neurocognitive, pulmonary and cardiac symptoms. When symptoms after acute COVID-19 exceed 12 weeks, the National Institute for Health and Care Excellence (NICE) defines the symptom complex as “Post-COVID-19 syndrome” [1]. The most common symptoms reported by patients are fatigue, dyspnea, myalgia and chest pain [2]. However, the puzzle behind the pathophysiological mechanisms remains unsolved. Persistent inflammation, induced autoimmunity and viral persistence in the body are discussed as potential drivers [3]. Interestingly, even patients who suffered from a mild or moderate acute disease can develop long-lasting symptoms [4,5].

It is known that infectious diseases, especially viral diseases, such as Epstein–Barr virus (EBV) [6], severe acute respiratory syndrome (SARS) in 2003 [7], and the West Nile virus [8], can give rise to long-lasting symptoms. Recovery times vary between individuals and diseases. For example, approximately 10% of individuals have persistent fatigue six months after symptom onset of infectious mononucleosis [9], whereas up to 30% of people with West Nile virus infection have postviral fatigue with an average duration of 5 years [8]. Irrespective of the cause, fatigue is an important factor for quality of life and patients with diagnosed chronic fatigue syndrome showed remarkably lower scores in physical and mental dimensions of HRQoL [10].

Recently, our research group showed that physical- and mental-health-related quality of life (HRQoL) is substantially impaired in patients suffering from post-COVID-19 syndrome after a mild or moderate disease compared with the pre-pandemic general Swiss population [11]. A literature screening review found that at a follow-up at 12 weeks, the median estimate of non-hospitalized patients with ongoing symptoms is approximately 12% (7.5–41%) [12]. The disabilities due to symptoms might come with great economical loss considering the vast amount of affected people and all the potential excessive work absences due to the illness. 

Currently, researchers and clinicians lack knowledge about the course of post-COVID-19 symptoms and treatment options are scarce. Our clinical experience suggests that ongoing symptoms might subside over time. Tran et al. demonstrated that the prevalence of most post-COVID-19 symptoms decreases over time before plateauing 6–8 months after onset [13]. However, the evolution of the impact of post-COVID-19 symptoms on HRQoL after mild or moderate acute disease over time has not been thoroughly investigated. Therefore, we followed up on patients and aimed to characterize changes in HRQoL 6 months after an initial assessment.

## 2. Materials and Methods

### 2.1. Study Design and Patient Population

The departments of Pulmonology, Cardiology, Neurology and Internal Medicine at the University Hospital Zurich developed an interdisciplinary outpatient clinic for patients suffering from persistent symptoms after developing COVID-19. Questionnaires regarding HRQoL (St. George’s Respiratory Questionnaire (SGRQ), EuroQol 5 Dimension 5 Level (EQ-5D-5L) and Short Form-36 (SF-36)) were distributed to the patients during their visit to the outpatient clinic (baseline). For patients who completed the questionnaires at baseline, the same questionnaires were sent by letter to them after six months for a follow-up assessment. Additionally, patients received a questionnaire regarding COVID-19 vaccination at follow-up.

Inclusion criteria were properly completed questionnaires and patients who suffered from ongoing symptoms after developing confirmed or highly suspected acute mild or moderate COVID-19 without hospitalization. Mild illness was defined as any of the various symptoms of COVID-19 (e.g., fever, cough, sore throat, malaise, headache, muscle pain, nausea, vomiting, diarrhea, and loss of taste and smell) but without shortness of breath, dyspnea or abnormal chest imaging. Moderate illness was defined by clinical or radiological evidence of lower respiratory tract involvement but normal oxygen saturation (SpO_2_ ≥ 94%) with room air. Exclusion criteria were initial severe acute COVID-19 requiring prolonged hospitalization or intensive care treatment and patients with symptoms that were assigned to another diagnosis (e.g., asthma). 

Information about demographics, symptoms during acute infection and post-COVID-19 symptoms were drawn from systematically documented medical reports. Pre-existing asthma; pre-pandemic mental health issues; and cardiovascular, rheumatological and thyroid diseases were assessed as comorbidities. 

This study was conducted in accordance with the declaration of Helsinki and all subjects provided written informed consent via general consent. The Ethics Committee of the Canton of Zurich approved the study (BASEC 2021-00280), and the study is registered on www.ClinicalTrials.gov as NCT04793269 (accessed on 2 May 2023).

### 2.2. Questionnaires 

All patients received three different questionnaires regarding HRQoL, as well as one self-constructed questionnaire about the COVID-19 vaccination (see Supplementary Materials). 

#### 2.2.1. St. George’s Respiratory Questionnaire

The St. George’s Respiratory Questionnaire (SGRQ) is a validated quality of life assessment tool used to evaluate the impact of respiratory symptoms on everyday life [14,15]. The symptom frequency and severity of respiratory symptoms are measured, and limitations in activity, as well as the social and emotional impacts, due to the disease are covered. Each item is weighted according to the degree of distress. Scores range from 0 to 100, with higher scores indicating worse quality of life. Missing items were handled according to the SGRQ manual [16]. An improvement of 4 points is accepted as the minimal clinically important difference (MCID) in the literature [17].

#### 2.2.2. EuroQol 5 Dimension 5 Level 

The EuroQol 5 Dimension 5 Level (EQ-5D-5L) is widely used as a generic measure of health status [18]. The first part (the descriptive system) comprises five dimensions, namely, mobility, self-care, usual activities, pain/discomfort and anxiety/depression. For every dimension, patients are asked to assign a level of severity, ranging from 1 “no problems” to 5 “extreme problems”. Patients’ responses are then combined to produce a five-digit number describing the participant’s health status. Each health state can potentially be assigned a summary index score based on societal preference weights for the health state. Index scores range from less than 0 (dead) to 1 (full health). Index scores were calculated using Germany-specific value sets as we judged the population of Germany to be comparable to the Swiss German population. In the second part of the questionnaire, the self-rated health of patients was recorded using a visual analog scale (VAS) ranging from 0 (worst health) to 100 (best health). The minimally important difference for the EQ index value ranges between 0.03 and 0.069 points [19,20]. For EQ, VAS scores with a difference of 5.0 are suggested to show MCID in fibrotic interstitial lung disease [21].

#### 2.2.3. Short Form-36

The Short Form Health 36 (SF-36) is a multidimensional instrument for measuring HRQoL [22]. It includes eight health dimensions that evaluate physical problems, role limitations due to physical problems, pain, general health, vitality, social functioning, role limitations due to emotional problems and mental health. The health dimensions consist of the summed scores of the assigned questions. Scores range from 0 (worst possible health) to 100 (best possible health). All health dimensions contribute in different proportions to create two summary score components: the physical component summary (PCS) and the mental component summary (MCS). Out of the health dimension scores, a z-score is determined for each dimension by subtracting the dimension mean of the U.S. population from an individual’s dimension score and dividing it by the standard deviation from the U.S. general population [23]. Each of the eight z-scores is multiplied by the corresponding factor scoring coefficient (separately for PCS and MCS) for the dimension [24]. Products of the z-scores are summed together, multiplied by 10, and added to 50 to linearly transform the PCS and MCS to T-score metrics. A value of 50 for the norm-based score represents the mean of the respective reference population and higher values mean better HRQoL. For the PCS and MCS T-scores, a 3-point change is suggested for an MCID [25]. Bjorner et al. recommended an MCID of 5 points for the health dimension vitality [26].

#### 2.2.4. Questionnaire about COVID-19 Vaccination

This self-created questionnaire was used to assess the subjective effect of the vaccination on post-COVID-19 symptoms. Besides questions assessing the type, date and adverse events of the vaccine, patients were asked to rate whether the vaccine led to an improvement or worsening of post-COVID-19 symptoms and whether the change in symptom severity was persistent. Moreover, the overall improvement or worsening of symptoms could be displayed using a visual analog scale from 0 (no improvement) to 10 (best possible improvement/no more symptoms).

### 2.3. Statistical Analysis

Descriptive statistics of baseline patient characteristics are presented as the mean and standard deviation (SD) or median and 25%/75% quartiles (quartiles) for continuous measurements and as the number and percentage of total for categorical measurements. Changes in HRQoL were compared using a paired *t*-test and Wilcoxon signed rank test for continuous variables. Multivariable regression analysis was performed to test for possible predictors (i.e., sex and subjective effect of vaccination). Missing data were not replaced. All statistical tests were two-tailed and a *p*-value of <0.05 was considered statistically significant. Statistical analysis was performed using Stata version 16.1 (StataCorp. 2019, College Station, TX, USA). No a priori sample size calculation was performed due to the exploratory study design.

## 3. Results

### 3.1. Study Sample

**Figure 1:**
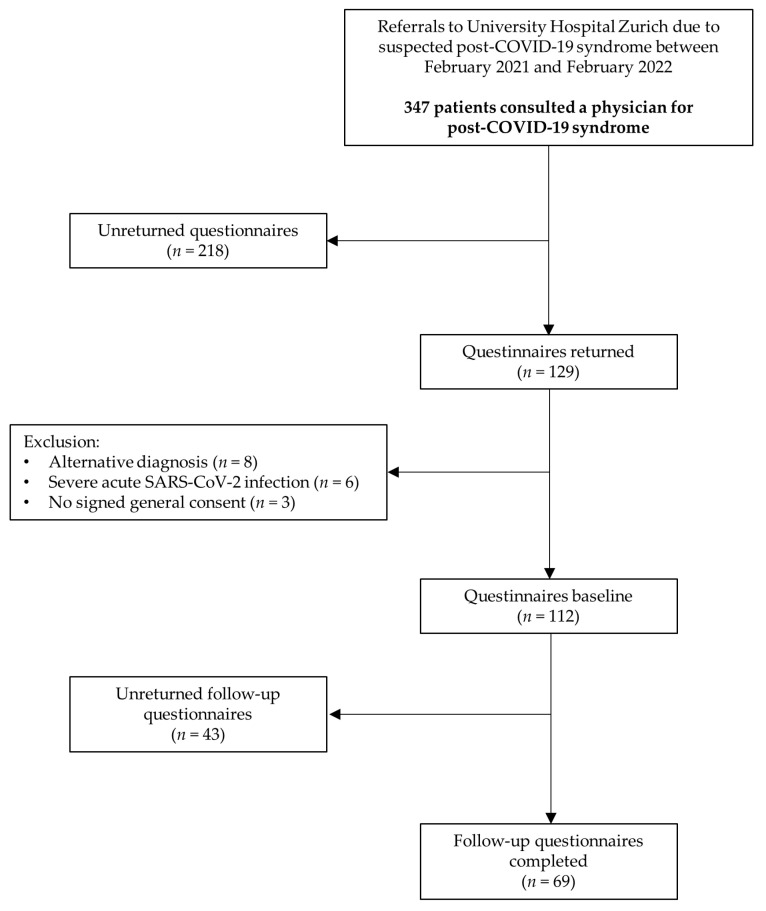
Study flow.

In this observational follow-up study, 112 patients with post-COVID-19 syndrome completed questionnaires at baseline, of whom 69 also completed follow-up questionnaires (38.4% lost to follow-up) (Figure 1). Patients at follow-up did not differ significantly with regard to physical HRQoL in the SF-36 from patients who were not followed up on (PCS mean (95% CI) difference of 1.2 (−2.8, 5.3) points, *p* = 0.551). However, patients who did not complete the follow-up had significantly lower mental HRQoL in the SF-36 compared with patients who completed the follow-up (MCS mean (95% CI) difference of –4.9 (−9.4, −0.4) points, *p* = 0.032). Subjects were predominantly female (80.0%) with a mean (SD) age of 44 (11.9) years. The median (IQR) score for the body mass index was 24.2 (21.5, 26.7) kg/m^2^. The median (IQR) time from symptom onset to completing the follow-up questionnaires was 326 (300, 391) days. Before the pandemic, nine (13.0%) patients suffered from asthma, 7 (10.1%) had mental health issues and 14 (20.3%) had at least one relevant comorbidity. The majority of patients (94.2%) suffered from a mild initial COVID-19. Approximately one-third of the patients stated to work less because of long-lasting COVID-19 symptoms. Further patient characteristics are outlined in Table 1.

### 3.2. Symptom Characterization at Baseline

At baseline, patients mostly stated having neurocognitive symptoms, such as fatigue (75.4%) and concentration difficulties (56.5%), as well as cardiorespiratory problems, including dyspnea (59.4%), performance intolerance (55.1%) and thoracic pain (50.7%). See Table S1 (Supplementary Materials) for all symptom frequencies. 

### 3.3. Subjective Effect of Vaccination on Post-COVID-19 Symptoms

A total of 97.1% of patients who completed the follow-up received a COVID-19 vaccine. A median (quartiles) time of 192 (147, 242) days passed from the onset of acute symptoms of COVID-19 to the first shot of the vaccine. About half of the patients received only one vaccine shot. There were 27 patients (40.3%) who had the impression of a persistent improvement of symptoms after a median (quartiles) time of 2 (1, 4) weeks after their vaccination. A persistent worsening of symptoms was stated by 22.4% of patients after a median (quartiles) time of 1 (1, 4) week, and 29.9% of patients neither felt an improvement nor a worsening of symptoms after receiving the vaccine (Table S2, Supplementary Materials).

### 3.4. SGRQ Questionnaire

Overall, all SGRQ component scores improved significantly after a follow-up of 6 months (Table 2). The largest mean (95% CI) difference of −14.4 (−18.4, −10.3) points was reached in the symptoms score component, whereas the lowest mean (95% CI) difference scores were reached in the impact scores component, with −6.5 (−10.2, −2.7) points. The SGRQ total score component showed a mean (95% CI) difference of –9.4 (−13.3, −5.5) points. 

### 3.5. EQ-5D-5L

**Figure 2:**
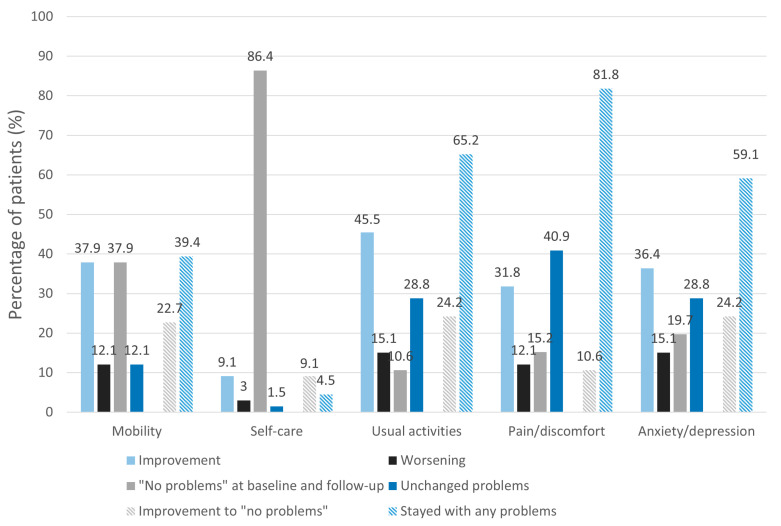
Changes in EQ-5D-5L dimension responses. The figure shows the changes from baseline (improvement/worsening/“no problems” at baseline and follow-up/unchanged problems) in the percentage of patients in the health dimensions of mobility, self-care, usual activities, pain/discomfort and anxiety/depression of the Euroqol-5D-5L questionnaire (EQ-5D-5L). Furthermore, percentages of patients that improved to “no problems” or stayed with any problems are shown. In almost all dimensions (with the exception of self-care), patients stated an improvement. Patients mostly did not report any problems with self-care at baseline, as well as at follow-up.

Figure 2 shows the percentage of patients with changes in the dimensions of mobility, self-care, usual activities, pain/discomfort and anxiety/depression. With the exception of the self-care and pain/discomfort dimensions, the majority of patients improved in all EQ-5D-5L health dimensions. At least one in five patients (20%) had an improvement to the level of “no problems” in the dimensions of mobility, usual activities and anxiety/depression. Self-care was the dimension where patients mostly stated having “no problems” at baseline and follow-up. Lower scores at follow-up visits were stated by 15% in the usual activities and anxiety/depression dimensions, 12% in the mobility and pain/discomfort dimensions, and 3% in the self-care dimension. However, there was a significantly greater proportion of patients with improvements compared with worsening in all dimensions, except in the dimension of self-care, where most patients stated having “no problems” at all (*p* = 0.003 for mobility, *p* = 0.002 for usual activities, *p* = 0.016 for pain/discomfort, *p* = 0.016 for anxiety/depression, *p* = 0.157 for self-care). Almost 50% of patients who suffered from pain/discomfort had persistent difficulties in this dimension.

Dimension scores at baseline and follow-up are displayed in Figure S1 (Supplementary Materials).

Regarding the EQ index value, there was a statistically significant improvement (mean (95% CI) difference of 0.060 (0.019, 0.102), *p* = 0.005) between baseline and follow-up. Furthermore, patients had significantly higher mean (SD) EQ VAS scores at follow-up (59.1 (20.9) vs. 66.0 (20.3), *p* = 0.002) (Table 2).

### 3.6. SF-36

Mean (SD) scores and mean (95% CI) differences for the eight SF-36 health dimensions and PCS and MCS T-scores are outlined in Table 3. Patients improved significantly in the dimensions of physical functioning, physical role limitations, pain, energy/vitality, emotional role limitations and emotional health at follow-up. The mean (SD) scores of the physical component summary (PCS) score were significantly higher at follow-up (39.2 (10.2) vs. 43.0 (10.9), *p* < 0.001), whereas the mental component summary (MCS) score showed no significant change (41.8 (11.5) vs. 44.1 (11.5), *p* = 0.069). The mean (95% CI) difference scores between follow-up and baseline for the PCS and MCS were 4.9 (2.6, 7.2) and 2.3 (−0.2, 4.8), respectively. No significant difference was found in the dimensions of general health and social functioning between baseline and follow-up. Sex, the subjective effect of the COVID-19 vaccine on post-COVID-19 symptoms, the time from the symptom onset of COVID-19 to the first vaccination and the number of vaccine shots were not independent predictors for the change in the PCS or MCS.

## 4. Discussion

This study investigated the change in health-related quality of life in patients suffering from long-lasting symptoms after mild or moderate acute COVID-19 over time. We found that physical HRQoL, including several aspects of daily living, relevantly improved 6 months from baseline despite treatment options being scarce.

Recently, our research group showed that physical and mental health are substantially impaired in patients referred for a post-COVID-19 consultation compared with the Swiss general population during pre-pandemic times [11]. In particular, the health dimensions “usual activities”, “pain” and “anxiety/depression” were affected, whereas “self-care” did not seem to be impaired at all. In patients who were hospitalized due to acute COVID-19, most individuals still reported symptoms 12 months after hospitalization [27]. After an initial mild disease, studies showed persistent symptoms also in this patient group [28,29]. Apart from symptom persistence, little is known about the consequences on HRQoL over time in patients with post-COVID-19 symptoms who suffered an initial mild or moderate COVID-19.

Regarding physical health, patients showed statistically significant and clinically relevant improvements exceeding the recommended 3-point minimal clinically important difference over six months. This was also reflected in the EQ-5D-5L, as one in five patients had improvements to “no problems” in the health dimensions “mobility” and “usual activities”, and therefore, this seems to demonstrate potentially higher activity levels in those patients. Our results are contradictory to the findings of Seessle et al. [30], who found decreased physical HRQoL in patients with mild or moderate disease 12 months after the acute disease. This might have been due to the reason that their study cohort consisted of patients, which were considerably older and more patients suffered from an initial moderate disease severity (55.2% vs. 5.8%). Although the pathophysiological mechanisms that might have led to an improvement in symptoms are not available and specific therapies are still missing, physical health relevantly improved over time. It is difficult to say whether time was the key factor for the improvement of physical HRQoL or whether patients learned to cope with their illness, and therefore, did not feel as restricted physically. The considerable proportion of patients (40%) who stated that they had a persistent reduction in symptoms after the COVID-19 vaccine indicated that the severity and/or number of symptoms reduced over time. Complementary to that, Ayoubkhani et al. [31] observed a considerable likelihood of post-COVID-19 symptoms decreasing after COVID-19 vaccination. Further, the SGRQ’s symptoms component score, which represents the frequency and severity of respiratory symptoms, showed significantly improved scores. Due to missing evidence-based treatment options, patients try various self-administered or experimental treatment strategies, as well as in- and outpatient rehabilitation programs. Therefore, it is unknown whether and to what extent those therapies or lifestyle changes, such as pacing, also influenced the course of physical HRQoL in a positive way. 

No significant improvement was observed in mental health. Mental health might take more time to improve compared with physical health, and thus, the follow-up of 6 months could have been insufficient to detect a significant change. However, as we showed in our previous study [11], mental health was similarly impaired in post-COVID-19 patients and a control group during the first wave of the pandemic compared with the general Swiss population before the pandemic. Subsequently, mental health deterioration might have evolved as a consequence of socio-economical and political changes during the pandemic, and therefore, affect the whole population and not only post-COVID-19 patients. Lastly, it was already reported that physical disabilities might lead to depression [32], and thus, the long period of one year that patients had been suffering from post-COVID-19 symptoms in our cohort might have contributed to persistent mental health issues. Approximately 60% and 45% of patients in the dimensions “pain/discomfort” and “anxiety/depression”, respectively, showed a decline or no change, which also might explain the non-significant improvement in mental health. 

Future studies are warranted to investigate predictors for improvement or worsening in physical and mental HRQoL so that the course of the disease and its impact on different patient groups can be better understood. In our study cohort, sex, the subjective effect of the SARS-CoV-2-specific vaccination after infection on post-COVID-19 symptoms, the time from symptom onset of COVID-19 disease to the first vaccination, and the amount of vaccine shots were not independent predictors for physical or mental health. 

Patients with follow-up showed a higher mental health status at baseline compared with individuals without follow-up. We cannot tell whether these patients did not participate due to remaining impairments or whether other reasons, such as motivational issues, hindered them. However, physical health at baseline was comparable in patients with and without follow-up. Moreover, the response rate to follow-up questionnaires was 61.6%, which can be rated as high enough. There is little literature regarding response rates to follow-up questionnaires, but one randomized trial that compared response rates with and without incentives showed similar results (68.5%) to the group where no incentives were given [33]. 

This study had some limitations. It is difficult to differentiate what effect can be allocated to time alone and what could be allocated to patients’ self-effort or treatment strategies. However, the focus of this study was primarily to assess the change in HRQoL. Additional studies are needed that investigate the course of symptoms over time, as well as treatment strategies on the impact of HRQoL. A previous sample size calculation was not performed due to the exploratory study design, and therefore, the sample size was too small to test for various predictors. However, since the mean PCS was above the MCID and the lower limit was near the MCID, we concluded that our findings have enough power. Further, well-powered studies with bigger sample sizes should be conducted to confirm the findings and to evaluate possible predictors and the influence of different COVID-19 variants on the course of post-COVID-19. Lastly, as this study depicted only a patient collection of the German-speaking part of Switzerland with a generally very stable political situation and labor market, it is difficult to apply our results to other regions of the world. 

## 5. Conclusions

The majority of patients that initially suffered from mild or moderate acute COVID-19 showed significant and clinically relevant improvements in physical-health-related quality of life over 6 months. Future studies are needed to better understand the course of the disease in different patient groups and whether the findings persist over time.

## Figures and Tables

**Table 1:** Patient characteristics.

	*n* = 69
Sex	
Female	55 (80.0)
Male	14 (20.0)
Age, mean (SD)	44.2 (11.9)
BMI kg/m^2^, median (IQR)	24.2 (21.5, 26.7)
WHO classification	
Mild	65 (94.2)
Moderate	4 (5.8)
Days from first questionnaire to follow-up questionnaire, median (IQR)	182 (174, 192)
Days from symptom onset to follow-up questionnaire, median (IQR)	326 (300, 391)
Smoking history	
Current	6 (8.7)
Former	18 (26.1)
Never	38 (55.2)
Ethnicity	
Caucasian	58 (84)
Not Caucasian	2 (3)
Missing data	9 (13)
Marital status	
Living with a partner	38 (55.1)
Living alone	10 (14.5)
Missing data	21 (30.4)
Reduced employment due to post-COVID-19	22 (31.9)
Reduced ≥ 50%	10 (14.5)
Reduced < 50%	12 (17.4)
Comorbidities	
Asthma	9 (13.0)
Prepandemic mental health issues	7 (10.1)
Other relevant comorbidities ^a^	14 (20.3)

Values are *n* (%) unless otherwise stated. ^a^ Other relevant comorbidities were relevant cardiovascular disorders, rheumatological diseases and diseases of the thyroid.

**Table 2:** SGRQ component scores, EQ-5D-5L index value and EQ-5D-5L VAS scores.

	Baseline, Mean (SD)	Follow-Up, Mean (SD)	Δ *, Mean (95% CI)	*p*-Value
SGRQ symptom score ^a^	40.4 (21.0)	26.0 (21.1)	−14.4 (−18.4, −10.3)	*p* < 0.001
SGRQ activity score ^b^	51.3 (25.4)	39.1 (27.6)	−12.2 (−17.4, −7.0)	*p* < 0.001
SGRQ impact score ^b^	27.2 (17.7)	20.8 (18.5)	−6.5 (−10.2, −2.7)	*p* = 0.001
SGRQ total score ^c^	37.3 (20.2)	27.9 (20.2)	−9.4 (−13.3, −5.5)	*p* < 0.001
EQ index value ^d^	0.758 (0.203)	0.818 (0.168)	0.060 (0.019, 0.102)	*p* = 0.005
EQ VAS ^e^	59.1 (20.9)	66.0 (20.3)	6.9 (2.7, 11.1)	*p* = 0.002

^a^ *n* = 69, ^b^ *n* = 57, ^c^ *n* = 51, ^d^ = 66, ^e^ = 67. SGRQ: lower scores mean better quality of life. EQ index value and EQ VAS: higher scores mean better quality of life. * Change from baseline.

**Table 3:** SF-36 health domain scores and PCS and MCS T-scores.

	Baseline, Mean (SD)	Follow-Up, Mean (SD)	Δ *, Mean (95% CI)	*p*-Value
Physical functioning ^a^	63.4 (24.6)	75.4 (20.3)	12.1 (7.0, 17.1)	*p* < 0.001
Role limitations (physical) ^b^	27.6 (35.7)	48.1 (42.9)	20.5 (10.9, 30.2)	*p* < 0.001
Pain ^b^	56.7 (28.9)	67.3 (28.7)	10.7 (4.2, 17.1)	*p* = 0.002
General ^c^	54.1 (18.2)	54.7 (19.1)	0.7 (−3.7, 4.9)	*p* = 0.795
Energy/vitality ^c^	29.8 (19.4)	40.8 (21.7)	11.0 (6.3, 15.7)	*p* < 0.001
Social functioning ^d^	57.9 (28.2)	62.5 (29.7)	4.6 (−2.4, 11.7)	*p* = 0.195
Role limitations (emotional) ^b^	57.7 (44.8)	69.2 (41.2)	11.4 (0.98, 21.9)	*p* = 0.032
Emotional health ^c^	61.3 (19.1)	66.9 (18.0)	5.6 (1.3, 9.8)	*p* = 0.011
PCS (T-score) ^d^	38.2 (10.2)	43.0 (10.9)	4.9 (2.6, 7.2)	*p* < 0.001
MCS (T-score) ^d^	41.8 (11.5)	44.1 (11.5)	2.3 (−0.2, 4.8)	*p* = 0.069

^a^ *n* = 68, ^b^ *n* = 67, ^c^ *n* = 66, ^d^ *n* = 65. * Change from baseline. SF-36 scores: higher scores mean better quality of life.

## Data Availability

The data that support the findings of this study are available on request from the corresponding author.

## Supplementary Materials

The following supporting information can be downloaded from https://www.mdpi.com/article/10.3390/jcm12124077/s1: Figure S1: EQ-5D-5L dimension responses at baseline and follow-up; Table S1: Prevalence of long COVID symptoms; Table S2: Effect of COVID-19 vaccine on long COVID symptoms.
