# Supplementary material for: Physical Health-Related Quality of Life Improves over Time in Post-COVID-19 Patients: An Exploratory Prospective Study
Source: J Clin Med. 2023 Jun 15;12(12):4077. doi: 10.3390/jcm12124077 (PMC10298963; doi:10.3390/jcm12124077)
Supplement: Supplementary file 1 [file jcm-12-04077-s001.zip › Malesevic_QoL in Post-COVID_Supplements.pdf]

Journal of
Clinical Medicine

Article

Physical Health-Related Quality of Life Improves over Time in Post-COVID-19 Patients: An Exploratory Prospective Study—Supplementary Materials

Stefan Malesevic [1,2], Noriane A Sievi [2], Dörthe Schmidt [3], Florence Vallelian [4], Ilijas Jelcic [5], Malcolm Kohler [2] and Christian F Clarenbach [2,*]

[1] Faculty of Medicine, University of Zurich, 8006 Zurich, Switzerland; stefan.malesevic@usz.ch
[2] Department of Pulmonology, University Hospital Zurich, 8091 Zurich, Switzerland
[3] Department of Cardiology, University Hospital Zurich, 8091 Zurich, Switzerland
[4] Department of Internal Medicine, University Hospital Zurich, 8091 Zurich, Switzerland
[5] Department of Neurology, University Hospital Zurich, 8091 Zurich, Switzerland
* Correspondence: christian.clarenbach@usz.ch

Citation: Malesevic, S.; Sievi, N.A.; Schmidt, D.; Vallelian, F.; Jelcic, I.; Kohler, M.; Clarenbach, C.F. Physical Health-Related Quality of Life Improves over Time in Post-COVID-19 Patients: An Exploratory Prospective Study. *J. Clin. Med.* **2023**, *12*, 4077. https://doi.org/10.3390/jcm12124077

Academic Editors: César Fernández De Las Peñas and Domingo Palacios-Ceña

Received: 4 May 2023
Revised: 12 June 2023
Accepted: 13 June 2023
Published: 15 June 2023

Copyright: © 2023 by the authors. Licensee MDPI, Basel, Switzerland. This article is an open access article distributed under the terms and conditions of the Creative Commons Attribution (CC BY) license (https://creativecommons.org/licenses/by/4.0/).

Table S1. Prevalence of Post-COVID-19 symptoms.

Symptom	*n* (%)
Fatigue	52 (75.4)
Dyspnea	41 (59.4)
Concentration difficulties	39 (56.5)
Performance intolerance	38 (55.1)
Thoracic pain	35 (50.7)
Headache	30 (43.5)
Smell alteration	22 (31.9)
Dizziness	21 (30.4)
Sleeping difficulties	18 (26.1)
Muscle weakness	18 (26.1)
Palpitations	18 (26.1)
Cough	16 (23.2)
Muscle pain	15 (21.7)
Taste alteration	12 (17.4)
Memory difficulties	12 (17.4)
Gastrointestinal symptoms (either nausea, vomiting or diarrhea)	12 (17.4)
Weight gain	11 (15.9)
Weight loss	10 (14.5)
Sputum production	6 (8.7)
Temperature	5 (7.2)
Reflux	4 (5.8)
Joint pain	3 (4.3)
Orthopnea	3 (4.3)
Tingling paresthesia	3 (4.3)
Swallowing difficulties	2 (2.9)
Postexertional malaise	2 (2.9)
Hair loss	1 (1.4)

Table S2. Effect of COVID-19 vaccine on Post-COVID-19 symptoms**.**

COVID-19 Vaccine	N (%)
Received vaccination after initial SARS-CoV-2 infection	67 (97.1)
Moderna	47 (70.1)
Pfizer	19 (28.4)
Unknown	1 (1.5)
1 vaccination	35 (50.7)
2 vaccinations	28 (40.6)
2 vaccinations + Booster vaccination	3 (4.4)
Missing information about number of vaccinations	3 (4.4)
Days from symptom onset of acute COVID-19 until first vaccination, median (IQR)	192 (147, 242)
Effect on Long-Covid symptoms	
Improvement	34 (50.7)
Persistent improvement	27 (40.3)
Weeks until improvement, median (IQR)	2 (1, 4)
Worsening	18 (26.9)
Persistent worsening	15 (22.4)
Weeks until worsening, median (IQR)	1 (1, 4)
Whether improvement nor worsening	20 (29.9)

Values are *n* (%) unless otherwise stated. Multiple selection of answers was possible.

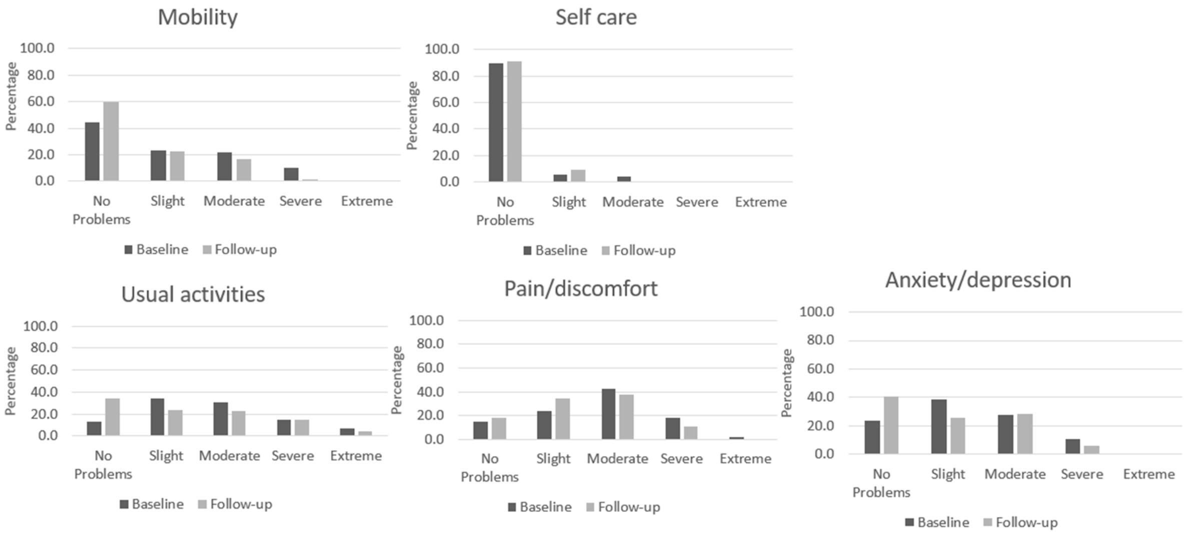

Figure S1. EQ-5D-5L dimensions responses at baseline and follow-up. Figure represents dimension responses as percentages of patients for the health dimensions mobility, self-care, usual activities, pain/discomfort and anxiety/depression of the Euroqol-5D-5L questionnaire (EQ-5D-5L). Response possibilities were "no problems", "slight problems", "moderate problems", "severe problems" and "extreme problems". The proportion of patients with the level "no problems" increases in all health dimensions.
